# Breeding for resilience to increasing temperatures: A field trial assessing genetic variation in soft red winter wheat

**DOI:** 10.1002/ece3.4668

**Published:** 2018-11-08

**Authors:** Kathleen Russell, David Van Sanford

**Affiliations:** ^1^ Department of Plant and Soil Sciences University of Kentucky Lexington Kentucky

**Keywords:** climate change, photoperiod sensitivity, resilience, wheat

## Abstract

Breeding for resilience to climate change is a daunting prospect. Crop and climate models tell us that global wheat yields are likely to decline as the climate warms, causing a significant risk to global food security. High temperatures are known to affect crop development yet breeding for tolerance to heat stress is difficult to achieve in field environments. We conducted an active warming study over two years to quantify the effects of heat stress on genetic variation of soft red winter (SRW) wheat (*Triticum aestivum* L.). Forty SRW cultivars and breeding lines were chosen based on marker genotypes at photoperiod sensitivity and reduced height loci. These genotypes were planted in a randomized complete block design replicated twice across two environments, ambient and artificially warmed. Average heading date occurred 5 days earlier in the warmed environment than in the ambient environment over both years (*p* ≤ 0.05). On average, grain yield was significantly reduced in the warmed environment by 211.41 kg/ha (*p* ≤ 0.05) or 4.84%, though we identified 13 genotypes with increased yield in response to warming in both years. Of these genotypes, eight had significantly increased N uptake while six showed significantly increased N utilization efficiency under warming. Under warming, genotypes with wild‐type alleles at the Rht‐D1 locus display significantly greater yields (*p* ≤ 0.01) and biomass (*p* ≤ 0.001) than genotypes with reduced height alleles. Of the 13 genotypes with higher (*p* ≤ 0.01) yields under warming, nine have the wild‐type allele at the Rht‐D1 locus in addition to being photoperiod insensitive. The next steps will be to validate these findings in other populations and to develop an efficient breeding/phenotyping scheme that will lead to more resilient cultivars.

## 1 INTRODUCTION

Breeding for resilience to climate change is a daunting prospect. To begin with, it has been suggested that the distribution of crops will be shifted by the effects, direct and indirect, of climate change. An example is the predicted decline of species that control crop pests that will impact agriculture in Europe (Pecl et al., 2017). Further, Hatfield, Wright‐Morton, and Hall (2018) argue that new synergies between agronomists, geneticists, and agricultural meteorologists must be fashioned if the midwestern United States is to maintain a robust grain production system. Beyond these big picture concerns, at the level of focus of the individual wheat breeder, while it is likely that a warmer climate will result in lower wheat yields (Asseng, Foster, & Turners, 2011; Zhao et al., 2017), it is not clear which traits should be the object of a breeding focus. Tack, Barkley, Rife, Poland, and Nalley (2016) stated that future breeding efforts should focus on differential response of individual breeding lines to increased heat. Semenov, Stratonovitch, Alghabari, and Gooding (2014) recommended increased focus on heat and drought resistance in future breeding efforts, while Reynolds et al. (2016) argued that breeding efforts should be targeted toward adaptation to climate change, utilizing contrasting genotypes in stress environments. Chapman, Chakraborty, Dreccer, and Howden (2012) note the need to invest in the identifying and characterizing of adaptive traits that are important in breeding for climate change resilience. Further, they state adaptation to higher temperatures will be a key breeding strategy.

While other investigators have used artificially warmed environments in screening wheat varieties, the focus of those studies has not been to quantify genetic variation, but rather to assess physiological traits in depth (Ottman, Kimball, White, & Wall, 2011; Tian et al., 2014). It is essential that we identify lines that show heat tolerance that may be used as parents to generate populations from which even more heat tolerant lines can be selected.

The Intergovernmental Panel on Climate Change (IPCC) projects that temperature in the United States will increase 2–3°C (IPCC 2014) in the coming decades. In the southeastern United States, while there is considerable year to year temperature variability, a general warming trend has been observed (Foster, 2012). The most recent decade (2000–2010) was the warmest on record for the region. Projected temperature increases for the region indicate a potential annual increase in temperature of 5°C by 2100, with summer months expected to experience the greatest warming (Kunkel et al., 2013).

Temperature effects on crop growth and development are well documented (Asseng et al., 2011; Hatfield & Prueger, 2015; Moot, Henderson, Porter, & Semenov, 1996; Wahid, Gelani, Ashraf, & Foolad, 2007). The specific effect of temperature on development varies and may depend on circumstances such as duration, range from optimum, timing during development, and specific adaptations such as facultative photoperiodism (Wahid et al., 2007). Increased temperatures pose a serious threat to crop production globally; Asseng et al. (2015) observed through simulation experiments linked to field data that for every 1°C increase in mean temperature the reduction in wheat yield would range from 4.2% to 8.2%.

Selection for temperature stress tolerance in a field environment can be difficult due to variability of climate stress from season to season. This phenomenon is reflected in long‐term phenological datasets that demonstrate trends associated with changes in climate, particularly the earlier onset of spring since the 1960s and shifts in species distribution (Walther et al., 2002). Reynolds et al. (2016) suggested a need for targeted breeding efforts toward adaptation to climate change utilizing contrasting genotypes in stress environments. Ceccarelli et al. (2010) proposed selection for adaptation to climate change based on photoperiod (PPD) and temperature response that would allow stress avoidance at critical periods of development. Wheat photoperiod sensitivity is controlled by three genes (*Ppd‐A1*,* Ppd‐B1, and Ppd‐D1*) that map to the group 2 chromosomes (Kumar et al., 2012). Jones et al. (2017) suggest the *Ppd‐D1a* locus to be the most important driver of flowering time and duration in warmer environments; they observed a significant increase in flowering duration in cooler environments. Photoperiod insensitivity, along with duration and timing of flowering, has been proposed as selective strategies for earlier flowering to avoid excessive heat later in crop development (Jung & Müller, 2009).

In addition to PPD loci, height reducing alleles at the Rht loci have also been associated with the susceptibility to heat and drought stress (Alghabari, Lukac, Jones, & Gooding, 2014). While these alleles are widely deployed for reduced lodging (i.e., displacement from vertical growth), and increased yield in many regions, there is evidence that both *Rht‐B1b* and *Rht‐D1b* may be associated with increased sensitivity to drought and heat stress (Alghabari et al., 2014; Gale & Youssefian, 1985). In hot, dry conditions, the height reducing alleles at these loci have been implicated in reduced early vigor and shorter coleoptiles (the protective sheath on young shoots), in addition to reduced plant height (Gasperini et al., 2012; Rebetzke & Richards, 1999).

Stress tolerance has also been tied to resource‐use efficiencies. Tolerance in this context is described as the ability to compensate in an environment that is less suitable than normal, by utilizing available resources efficiently, despite a shift in phenology or shortened developmental period (Chapin, Bloom, Field, & Waring, 1987). Crop growth rate slows as temperatures increase above optimum temperatures, affecting photosynthesis and nitrogen (N) uptake (Hatfield et al., 2011). N uptake and remobilization of N are critical for wheat yield and grain quality. In wheat, up to 95% of the N that is partitioned to the grain is taken up during the vegetative period prior to anthesis (Gaju et al., 2014). Thus, N uptake and utilization along with rate of development are closely related to grain yield potential. Therefore, focusing on traits related to NUE can have potential adaptive benefits for yield potential under warming.

In the context of a breeding program, understanding the relationship between these adaptation strategies can lead to a more effective selection strategy for resilience to a changing climate. Previous research that involved artificial warming of wheat comprises studies primarily focused on physiological response rather than the genetic variation in traits that are suspected to control response to warming (Grant et al., 2011; Ottman et al., 2011; Zhao et al., 2016).

The objectives of this study were to (a) quantify variation among wheat cultivars and breeding lines in traits that are affected by warming; (b) assess the relationship between nitrogen use efficiency (NUE) and the ability to overcome heat stress in an artificially warmed environment; and (c) identify cultivars and breeding lines that maintain yield and quality under warming conditions.

## 2 MATERIALS AND METHODS

### 2.1 Site description and experimental design

The experimental material was a panel of 40 soft red winter (SRW) wheat cultivars and breeding lines adapted to the southeastern United States. Entries were selected on the basis of photoperiod sensitivity (*Ppd‐1*) alleles at the A1 and D1 loci determined using KASP genotyping chemistry (LGC, UK) analysis (Table 1). The study was grown over the 2015 and 2016 harvest years at the University of Kentucky Spindletop Research Farm in Lexington, KY (38°7′37.81″N, 84°29′44.85″W). The soil type at the site is a Maury silt loam soil [fine, mixed, semiactive, mesic Typic Paleudalfs soil].

**Table 1:** Panel of 40 soft red winter wheat genotypes with photoperiod and reduced height classification determined by marker analysis at two PPD and Rht loci^a^. These lines were tested under control and warmed environments, 2015–2016 growing seasons, Lexington, KY

Genotype	Photoperiod loci		Reduced height loci		
	Ppd‐A1	Ppd‐D1	Rht‐B1	Rht‐D1	
TRUMAN	Sensitive	Sensitive	b	a	
GA04121‐11E26	Sensitive	Sensitive	b	a	
BESS	Insensitive	Sensitive	a	b	*‡
NC08‐233324	Insensitive	Sensitive	a	b	
VA09W‐73	Insensitive	Sensitive	a	b	
LCS10516	Insensitive	Sensitive	b	a	
LCS19228	Insensitive	Sensitive	b	a	
LCS19229	Insensitive	Sensitive	b	a	
DANW1003	Insensitive	Sensitive	b	a	*‡
DANW1006	Insensitive	Sensitive	b	a	*†
DANW1008	Insensitive	Sensitive	a	b	*‡
AGS2000	Insensitive	Sensitive	b	a	
Pioneer 25R32	Insensitive	Sensitive	b	a	
GA041293‐11E54	Sensitive	Insensitive	a	b	
GA04434‐11E44	Sensitive	Insensitive	a	b	*‡
KY05C‐1121‐131‐3‐3	Sensitive	Insensitive	a	b	*‡
MD07W272‐11‐5	Sensitive	Insensitive	a	b	
Pioneer 26R61	Sensitive	Insensitive	a	b	
LA05038D‐105	Sensitive	Insensitive	a	b	*†
KY05C‐1381‐77‐17‐1	Sensitive	Insensitive	b	a	
MDC07026‐12‐10	Sensitive	Insensitive	a	b	
USG3555	Sensitive	Insensitive	a	b	
LA05130D‐P5	Sensitive	Insensitive	a	b	
PEMBROKE	Sensitive	Insensitive	a	b	
KY93C‐1238‐17‐1	Sensitive	Insensitive	b	a	*†
DINAH	Sensitive	Insensitive	a	b	
SS8700	Sensitive	Insensitive	b	a	*†
SSMPV57	Insensitive	Insensitive	b	a	
BRANSON	Insensitive	Insensitive	b	a	
PEMBROKE 2014	Insensitive	Insensitive	b	a	
SHIRLEY	Insensitive	Insensitive	b	a	*†‡
KWS011	Insensitive	Insensitive	b	a	*†
KWS013	Insensitive	Insensitive	a	b	
VA11W‐301	Insensitive	Insensitive	b	a	
JAMESTOWN	Insensitive	Insensitive	a	b	
PEMBROKE 2016	Insensitive	Insensitive	b	a	*†
AR00255‐16‐1	Insensitive	Insensitive	a	b	
KY05C‐1140‐8‐4‐1	Insensitive	Insensitive	a	b	
KY05C‐1105‐43‐6‐1	Insensitive	Insensitive	b	a	
OH07‐264‐35	Insensitive	Insensitive	b	a	*†

a Wild‐type allele (a), reduced height allele (b).

*Genotypes with positive yield response to warmed environment. †Genotypes with positive NUpE response to warmed environment. ‡Genotypes with positive NUtE response to warmed environment.

Planting dates were 24 October 2014 and 20 October 2015. The experiment was grown as a randomized complete block design with two replications and two treatments (hereafter referred to as ‘environments’). We were limited to two replications by the footprint of the warming study including warming cable length and headrow length. The experimental unit was a headrow (a general term related to a planting rate equal to the total number of seeds in one head), 1.5 m in length with a row spacing of 17.8 cm. The environments were a control (ambient) environment and an artificially warmed environment. Warming was achieved with five soil warming cables (Gro‐Quick 42 m length, 120V, 700 watt) buried at a depth of 2.54 cm between headrows to warm the rhizosphere within these plots. The temperature was monitored using two thermocouple wires (OMEGA Engineering, Stamford, Connecticut) placed 3 cm below the soil surface [within each of the plant rows per replication]. Thermocouple wires were also placed in the canopy 10 cm above each heating cable and attached to a stake to monitor air temperature in each block. The control block soil and air temperature were monitored in each replication for comparison. The thermocouple wire sensors were sampled at 15‐min intervals and averaged along each plant row. The sensors within plant rows on either side of each of the warming cables were averaged to determine the temperature threshold for each cable compared to the control plot. A CR1000 datalogger (Campbell Scientific, Logan, Utah) was located on site and programmed to activate the warming cables when the temperature sensors indicated less than a 5°C difference in temperature between the warmed and control environment. Warming began at early tillering (roughly one month post emergence). Two Watchdog 1000 Series WaterScout soil moisture probes (Spectrum Technologies, Aurora, Illinois) were placed within each environment to collect soil moisture data at 15‐min intervals from March to June when the soil was not frozen.

Nitrogen was applied as liquid urea ammonium nitrate (28%) in 2015 using a backpack sprayer (R&D Sprayers, Opelousas LA) and TeeJet flat fan nozzles (TeeJet Glendale Heights, IL) and as pelleted urea (46‐0‐0) in 2016. A total of 112 kg N/ha was applied in a 34 kg N/ha and 78 kg N/ha split on and 24 March and 13 April 2015 and as a single application of 112 kg N/ha on 24 March 2016 because weather conditions were not favorable for a split application.

### 2.2 Soil sampling

Soil samples were collected three times within each environment: prior to N application, at anthesis, and at physiological maturity. For each sampling, six soil cores were taken at a depth of 30.48 cm with a 1.6‐cm‐diameter soil probe. The cores were combined, air‐dried, and ground using a soil grinder.

Ammonium and nitrate were extracted from each soil sample using the KCl method: 2 mol KCl solution is prepared by diluting 150 g KCl in 1,000 ml of deionized water (Crutchfield & Grove, 2011). Ten grams of soil was combined with 25 ml of 2 mol KCl in 4 oz specimen cups. The solution was mixed for 30 min by shaking on a reciprocal shaker for 30 min at 200 rpm. One ml of solution was transferred to cluster tubes by pipette, and cluster tubes were centrifuged for 27 min. Aliquots (15 ml) of each sample and calibration standards were then pipetted into the wells of two microplates, one for the ammonium analysis and one for the nitrate analysis.

### 2.3 Agronomic traits and N sampling

Heading date was recorded when 50% of the plants in a headrow had visible spikes emerged from the flag leaf sheath. Anthesis date was recorded when 50% of the spikes had anthers extruding. Height was recorded at physiological maturity, and row length was measured just before harvest.

Each headrow was harvested at the soil surface after physiological maturity and plants were placed into paper bags to be air‐dried in the greenhouse. Head number and total weight were recorded for each headrow. Plants were threshed, and grain yield was measured. Vegetative biomass was determined by subtracting grain yield from the total plant weight. Harvest index was calculated as: grain yield (kg/ha)/grain yield (kg/ha) + vegetative biomass (kg/ha). Nitrogen harvest index was calculated as: grain N (kg/ha)/grain N (kg/ha) + vegetative N ((kg/ha). Spikes/m^2^ was determined by counting the overall number of heads per unit area.

Vegetative plant material from each headrow was ground to a powder using a cyclone mill (UDY One, Fort Collins, Colorado). Vegetative material and whole grain subsamples were analyzed for protein content using near‐infrared reflectance (NIR) on a DA7200 analyzer with a 950–1,650 nm wavelength range (Perten, Hägersten, Sweden). Grain protein content was divided by 5.7 to convert to N concentration (Osborne, 1907).

Total plant N uptake was determined by summing grain N (grain yield × % grain N) (kg/ha) and vegetative N at maturity (biomass yield × % vegetative N) (kg/ha). Nitrogen use efficiency (NUE) and its components, nitrogen uptake (NUpE) and utilization efficiency (NUtE) were calculated as: NUE = grain yield (kg/ha)/(total inorganic soil N + N applied (kg/ha)), NUpE = total plant N/total inorganic soil N (pre‐N soil N and N applied (kg/ha)), NUtE = yield/total plant N (Moll, Kamprath, & Jackson, 1982; Russell, Lee, & Van Sanford, 2017).

### 2.4 Statistical analysis

Analysis of variance (ANOVA) was performed using the general linear models procedure (Proc GLM; SAS 2011, Cary, North Carolina) to determine genotype and environment effects. The model used was:

$$Y_{\mathrm{ijkl}}=\mu +{\text{ENV}}_{i}+{\text{R(ENV)}}_{\mathrm{ij}}+{\text{YR}}_{k}+G_{l}+{\text{ENV}}_{i}\times G_{l}+{\text{ENV}}_{i}\times {\text{YR}}_{k}+{\text{YR}}_{k}\times G_{l}+E_{\mathrm{ijkl}}$$

where *Y*_*ijkl*_ = the observation in the *l*th genotype (G) in the *j*th rep (R) in the *i*th environment (ENV), in the *k*th year (YR), *μ* = the overall mean, R(ENV)_*ij*_ = the effect of *j*th rep within *i*th environment, ENV_*i*_ × *G*_*l*_ = the effect of the interaction of the *i*th environment and the *l*th genotype, and *E*_*ijkl*_ = the residual error. Least‐squares means were estimated for genotypes, environments, and genotype × environment combinations. Effects were considered significant at *p *≤* *0.05.

Heritability was estimated in each environment over the two years of the study by equating mean squares to their expectations, using the following linear model:

$$Y_{\mathrm{ijkl}}=\mu +{\text{YR}}_{i}+{\text{R(YR)}}_{\mathrm{ij}}+G_{l}+\text{YR}\times G_{l}+E_{\mathrm{ijkl}}$$

where terms are as defined above. Confidence intervals were estimated according to Knapp, Stroup, and Ross (1985).

## 3 RESULTS

### 3.1 Environment

**Figure 1:**
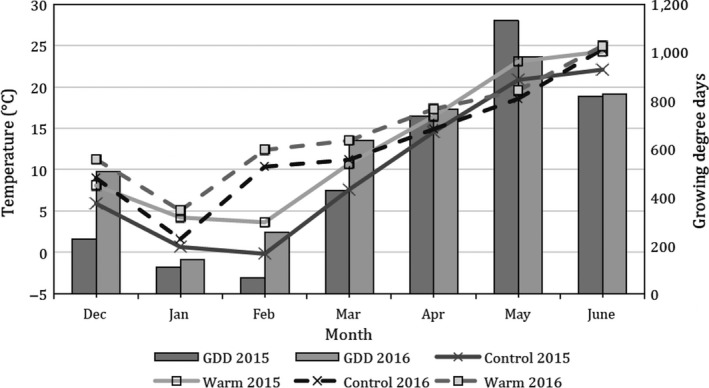
Average monthly soil temperatures and corresponding monthly accumulated growing degree days in actively warmed and control environments, 2014–2016 seasons, Lexington KY. 32°C base temperature for growing degree days (GDD)

The soil warming cables maintained an increase in soil temperature consistently throughout the growing seasons, creating an artificially warmed environment. While the datalogger was programmed for a 5°C temperature threshold, average soil temperatures in the warmed environment ranged from 1.48 to 3.81°C greater than the control environment (Figure 1). The two growing seasons varied considerably in monthly ambient temperature which influenced the temperature in the warmed environment. Growing degree days (GDD) are displayed by month for comparison (Figure 1).

**Figure 2:**
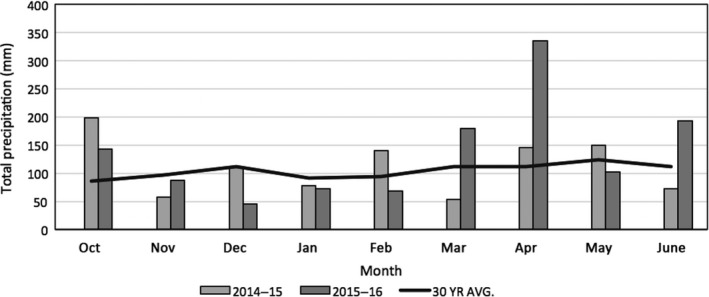
Total monthly precipitation and 30‐year average monthly precipitation, 2014–2016 seasons, Lexington KY

WaterScout soil moisture probes (Spectrum Technologies, Aurora, Illinois) showed no significant volumetric soil moisture content differences across environments across seasons. While the warming treatment was expected to cause soil drying, the placement of the soil warming cables in the upper 3 cm of the soil was offset by the precipitation that was not excluded in either environment (Figure 2). While the precipitation (snowfall) was below average in winter months (January–March) in both seasons compared to the 30 year average, the remainder of the season displayed greater than average precipitation which is consistent with the wheat growing season for the region (Figure 2).

### 3.2 Total inorganic soil N

There were no significant differences measured between environments for total inorganic soil N. Total inorganic soil N values in the warmed environment were slightly increased compared to the control environment at both the anthesis and maturity sampling. The warmed environment had 33.5 (±6.36) kg/ha compared to 29.3 (±5.75) kg/ha in the control environment for the 2014–2015 season at maturity. The warmed environment had 31.8 (±9.61) kg/ha compared to 20.64 (±3.49) kg/ha in the control environment in the 2015–2016 season at maturity.

### 3.3 Phenological traits

There were significant differences between environments for heading date (*p* ≤ 0.0001) and anthesis date (*p* ≤ 0.0001) with development accelerated in the warmed environment by 5 days (±0.07 days) on average across years (Table 2). Overall, genotypes headed and flowered earlier in 2016 than in 2015, but the phenology shift in response to warming was consistent across years.

**Table 2:** Mean agronomic trait response^a^ and standard error by environment, and *F* statistics for 40 soft red winter wheat genotypes from the combined ANOVA, Lexington, KY, 2015–2016. Sources, degrees of freedom (*df*), and significance levels for each term in the model are shown in the lower part of the table

Environment	*df*	Heading date (DOY ± Day)	Anthesis date (DOY ± Day)	Height (cm)	Spikes/m^2^	Yield (kg/ha)	Biomass (kg/ha)	Harvest index
Control		126 ± 0.07	128 ± 0.08	81.7 ± 0.26	493 ± 5.88	4,365 ± 67.45	7,031 ± 85.11	37.47 ± 0.31
Warmed		121 ± 0.07	124 ± 0.08	82.03 ± 0.26	450 ± 5.96	4,154 ± 67.88	6,735 ± 85.11	37.17 ± 0.31
E	1	1695.7***	1695.7***	0.76	27.14***	4.95*	6.05**	0.49
R[E]	2	104.7***	104.7***	5.4**	9.1***	7.59***	19.4***	2
Y	1	6575.48***	6575.48***	383.03***	169.18***	927.11***	735.63***	128.5***
G	39	15.9***	15.9***	31.25***	6.38***	4.31***	4.04***	3.18***
Y × E	1	0.01*	0.01*	155.41***	10.92***	0.01	3.42	2.22
G × Y	39	7.27***	7.27***	5.47***	3.59***	2.64***	2.05***	2.88***
G × E	39	2.34***	2.34***	3.64***	2.37***’	2.34***	2.17***	1.28

a Environment (E), rep (R), year (Y), genotype (G), day of year (DOY).

***p < 0.001,**p < 0.01,*p < 0.05.

### 3.4 Agronomic traits

Grain yield in the warmed environment was significantly (*p* ≤ 0.05) reduced by 4.8% or 211 kg/ha compared to the control environment with years combined (Table 2). Biomass also decreased significantly (*p* ≤ 0.01) by 4.2% or 296 kg/ha from the control environment to the warmed environment, averaged over years. Warming significantly (*p* ≤ 0.0001) reduced spike bearing stem number by 43 spikes/m^2^ (approximately 9%) compared to the control environment with a larger reduction in 2016 (Table 2).

Measured traits of interest were those that contribute to NUE, and thereby to heat stress tolerance, by maintaining the ability to remobilize assimilates effectively into the grain. In our high rainfall environment, the wheat crop is almost never subjected to moisture stress, in contrast to many areas where wheat is grown. Thus, while a warming environment is almost always associated with some sort of drought stress, it is not predicted to be the case in Kentucky and the mid‐south (Russell et al., 2017). Nitrogen, on the other hand, may be suboptimal at certain times during the crop year simply because of excessive moisture that leads to leaching and/or denitrification. In our study, when the two years were analyzed together, there were significant reductions in vegetative N content at maturity and total plant N content in the warmed environment (Table 3). Based on yield reductions, there was also a significant reduction in NUE in the warmed treatment. NUpE was significantly reduced (*p* ≤ 0.0001) but NUtE was not affected by the warming treatment (Table 3). This N response is interesting in that there were no differences in N content at anthesis between environments but there were significant reductions in TPN and vegetative N content at maturity in the warmed environment. The reduction in TPN can be explained in terms of vegetative N content because grain N content was not reduced in the warmed environment. Likely, the reduction in NUpE can be attributed to the shift in phenology and reduction in spikes/m^2^ in the warmed environment (Tables 2 and 3). Genotype × treatment interaction and year × treatment interaction were significant for most agronomic and nitrogen traits (Tables 2 and 3). Interaction least‐squares means and standard errors for these traits are presented in Supporting Information Table S1.

**Table 3:** Mean nitrogen trait response^a^ and standard errors by environment, and *F* statistics for 40 soft red winter wheat genotypes from the combined ANOVA, Lexington, KY, 2015–2016. Sources, degrees of freedom (*df*), and significance levels for each term in the model are shown in the lower part of the table

Environment	*df*	Vegetative nitrogen (Anthesis) (kg/ha)	Vegetative nitrogen (Maturity) (kg/ha)	Grain protein (%)	Grain nitrogen (kg/ha)	Total plant nitrogen (Maturity) (kg/ha)	NUtE (kg/kg)	NUpE (kg/ha)	NUE
Control		103.71 ± 3.31	41.26 ± 0.896	11.93 ± 0.04	85.8 ± 1.38	123.97 ± 1.83	34.37 ± 0.36	1.01 ± 0.01	34.01 ± 0.48
Warmed		109.69 ± 3.2	36.98 ± 0.9	11.83 ± 0.05	82.76 ± 1.42	116.65 ± 1.84	34.4 ± 0.36	0.94 ± 0.01	32.24 ± 0.49
E	1	1.75	11.33***	3.18	2.8	8.48**	0.23	15.32***	6.52**
R[E]	2	2.99*	10.54***	1.96	12.48***	18.03***	6.66***	14.35***	6.18**
Y	1	3.95*	0.81	2112.75***	396.69***	161.47***	905.36***	42.53***	217.44***
G	39	3.61***	2.29***	4.42***	3.46***	3.39***	2.64***	3.96***	4.79***
Y × E	1	0.59	12.72***	45.24***	3.68*	12.04***	13.45***	19.99***	0.19
G × Y	39	1.85	1.44*	3.35***	1.96***	1.86**	1.68*	2.13***	2.66***
G × E	39	0.79	1.94**	2.43***	2.57***	2.65***	2.03***	2.58***	2.23***

a Nitrogen harvest index (NHI), nitrogen utilization efficiency (NUtE), nitrogen uptake efficiency (NUpE), nitrogen use efficiency (NUE).

***p < 0.001,**p < 0.01,*p < 0.05.

Response of individual breeding lines to artificial warming varied over the two seasons. In 2016, there was a reduction in overall yield in both temperature environments compared to the previous season, yet we observed a consistent response in both years, with significantly reduced yields in response to warming. However, there was considerable genotypic variability in response. Thirteen entries had significant yield and NUE increases in the warmed environment when data from both years were combined (Table 1). Of these 13 genotypes, eight displayed significantly increased N uptake in the warmed environment compared to the control while six genotypes showed significantly increased N utilization efficiency in the warmed environment (Table 1). These components of NUE likely contributed to the increased yield in the warmed environment. In general, genotypes that responded with reduced yield in the warmed environment had greater N content in the biomass at maturity than in the control environment and thus had lower NUtE during the grain filling period.

### 3.5 Photoperiod response

*Ppd‐D1*‐sensitive genotypes had significantly delayed heading and anthesis dates in the warmed environment compared to the *Ppd‐D1*‐insensitive genotypes (Table 4) although shift in phenology remained delayed in the control environment. The earlier heading date in response to warming can also be viewed as a decline in overall time for vegetative growth. In PPD‐sensitive genotypes, this decline was 3.8% compared to 4.2% for PPD‐insensitive genotypes. Yield response of PPD‐insensitive genotypes to warming was significantly negative compared to PPD sensitive (*p* ≤ 0.02), while no significant decrease in yield was observed for PPD‐sensitive genotypes. Both PPD‐sensitive and PPD‐insensitive genotypes displayed significant small reductions in NUpE (Table 4).

**Table 4:** Mean response for agronomic and nitrogen traits^a^ and standard errors in control and actively warmed environments based on *Ppd‐D1* allele expression for a panel of 40 soft red winter wheat genotypes, two growing seasons, 2015–2016, Lexington, KY, calculated from the ANOVA

Photoperiod loci Ppd‐D1	Heading date (DOY)	Anthesis date (DOY)	Height (cm)	Yield (kg/ha)	Biomass (kg/ha)	NUtE (kg/kg)	NUpE (kg/ha)
Control environment							
Sensitive (*Ppd‐D1*)	127.75 ± 0.57	130.12 ± 0.57	84.84 ± 0.94	5226.28 ± 214.08	7880.76 ± 247.72	37.82 ± 1.2	1.01 ± 0.03
Insensitive (*Ppd‐D1a*)	126.84 ± 0.39	129.31 ± 0.39	80.79 ± 0.65	4666.0 ± 148.08	7382.61 ± 171.31	36.78 ± 0.83	0.97 ± 0.97
*p* value	NS	NS	***	*	NS	NS	*
Warmed environment							
Sensitive (*Ppd‐D1*)	123.2 ± 0.57	125.96 ± 0.59	88.41 ± 1.13	5056.4 ± 245.86	7495.72 ± 297.55	38.71 ± 1.13	0.97 ± 0.04
Insensitive (*Ppd‐D1a*)	121.74 ± 0.4	124.57 ± 0.41	81.84 ± 0.79	4448.43 ± 169.35	7239.01 ± 204.96	35.86 ± 0.77	0.91 ± 0.02
*p* value	*	*	***	*	NS	*	**

a Day of year (DOY), nitrogen utilization efficiency (NUtE), nitrogen uptake efficiency (NUpE). ***p < 0.001,**p < 0.01,*p < 0.05.

Of the genotypes with significant yield gains in the warmed environment both years: Four genotypes are PPD sensitive and nine are PPD insensitive at the *Ppd‐D1* locus (Table 1). Based on the full panel of genotypes tested, 31% of PPD sensitive (4 of 13) and 33% of PPD insensitive (9 of 27) had significant yield increases in the warmed environment. When comparing subsets of genotypes, we observed a significant increase in NUtE among PPD‐sensitive genotypes compared to the insensitive genotypes in the warming treatment (*p* ≤ 0.05) with no significant differences in NUpE (Table 4). This result contrasts with the analysis of the full panel of genotypes tested when grouped by PPD in Table 4.

Heritability estimates and 90% confidence intervals are presented in Table 5. In general, heritability estimates in the warmed environment were greater than those estimated under control conditions.

**Table 5:** Heritability estimates^a^ and 90% confidence intervals from a SRW wheat panel grown in control and actively warmed environments, Lexington, KY, 2015–16

Trait	Control environment			Warmed environment		
	*h* ^2^	LL	UL	*h* ^2^	LL	UL
HDOY	0.48	0.13	0.7	0.69	0.48	0.82
ADOY	0.32	−0.19	0.58	0.66	0.43	0.8
Height (cm)	0.74	0.57	0.87	0.84	0.73	0.9
Spikes/m^2^	0.35	−0.09	0.62	0.63	0.38	0.78
Harvest index	0.13	−0.46	0.49	0.13	−0.46	0.49
Yield (kg/ha)	0.38	−0.04	0.64	0.65	0.42	0.79
Biomass (kg/ha)	0.26	−0.24	0.56	0.74	0.56	0.85
Grain protein (%)	0.37	−0.06	0.63	0.33	−0.12	0.6
Grain N content (kg/ha)	0.29	−0.19	0.58	0.68	0.46	0.81
TPN (kg/ha)	0.31	−0.16	0.59	0.69	0.48	0.82
NUpE (kg/ha)	0.13	−0.48	0.48	0.63	0.37	0.78
NUtE (kg/kg)	0.31	−0.16	0.59	0.54	0.23	0.73
NUE	0.27	−0.23	0.56	0.64	0.39	0.79

Heading date of year (HDOY), anthesis date of year (ADOY), total plant nitrogen (TPN), nitrogen utilization efficiency (NUtE), nitrogen uptake efficiency (NUpE), nitrogen use efficiency (NUE).

### 3.6 Rht response

Under warmed conditions, genotypes with wild‐type alleles at the Rht‐D1 locus display significantly greater yields (*p* ≤ 0.01) and biomass (*p* ≤ 0.001) than genotypes with reduced height alleles (Table 6). Similarly, these genotypes also have significant increases in NUE and NUpE (*p* ≤ 0.001) (Table 6). Under ambient conditions, there were no significant differences among genotypes with Rht‐D1a (wild‐type) alleles and those with Rht‐D1b (height reducing) alleles which display a semi‐dwarf phenotype (Table 6). Of the 13 genotypes that showed significant yield response to warming across years, nine have the Rht‐D1a allele in addition to being PPD insensitive (Table 1).

**Table 6:** Entry means of traits^a^ measured under warming and control conditions and standard errors in 40 SRW wheat genotypes differing in the presence of reduced height alleles (Rht D1‐b) at the Rht D1 locus. Experiments were grown in Lexington, KY, 2015–2016

Reduced height loci	Spikes/m^2^	Yield (kg/ha)	Biomass (kg/ha)	Grain protein %	Grain N (kg/ha)
Control environment					
Rht D1‐b	465.5 ± 14.24	4639.7 ± 178.78	7400.4 ± 206.9	11.5 ± 0.18	90.9 ± 2.68
RhtD1‐a	472.4 ± 13.42	4972.8 ± 168.56	7704.2 ± 195.1	11.3 ± 0.17	92.4 ± 2.54
*p* value	NS	NS	NS	NS	NS
Warmed environment					
Rht D1‐b	400.7 ± 12.6	4104.5 ± 192.29	6628.8 ± 240.6	11.5 ± 0.15	81.6 ± 3.5
RhtD1‐a	460.8 ± 11.9	4984.2 ± 204.08	7909.1 ± 226.7	11.4 ± 0.14	97.6 ± 3.8
*p* value	***	**	***	NS	**

Reduced height loci	Total plant N (kg/ha)	NUtE (kg/kg)	NUpE (kg/ha)	NUE	Harvest index
Control environment					
Rht D1‐b	128.9 ± 3.08	35.7 ± 0.99	0.99 ± 0.02	34.2 ± 0.94	37.7 ± 0.51
RhtD1‐a	127.9 ± 2.92	37.6 ± 0.94	0.98 ± 0.02	36.3 ± 0.89	38.4 ± 0.48
*p* value	NS	NS	NS	NS	NS
Warmed environment					
Rht D1‐b	113.1 ± 4.16	35.6 ± 0.93	0.84 ± 0.03	29.8 ± 1.2	36.9 ± 0.62
RhtD1‐a	132.5 ± 4.43	37.2 ± 0.87	1 ± 0.03	36.7 ± 1.1	38.1 ± 0.65
*p* value	***	NS	***	***	NS

Nitrogen utilization efficiency (NUtE), nitrogen uptake efficiency (NUpE), nitrogen use efficiency (NUE). ***p < 0.001,**p < 0.01.

## 4 DISCUSSION

Temperatures that exceed optima at specific stages of crop development can significantly impact productivity (Hatfield & Prueger, 2015). Wheat yields are projected to decrease by 6.0% ± 2.9% with each 1°C increase in temperature based on models on a global scale (Zhao et al., 2017). Our experiment indicates that yield response to warming will be largely, though not necessarily uniformly, negative among wheat cultivars adapted to the southeastern United States. The in‐field screening method allowed for season‐long warming and exposure to typical field conditions (i.e., precipitation, pest pressures, cloud cover, mechanical traffic), which is not possible in controlled chamber experiments. Artificial warming experiments in wheat have focused on physiological traits without considering genetic variation in response to warming. Thus, this method also has novel applications for varietal improvement in the face of increasing temperatures. Further, active field warming data can be integrated with phenotypic data that is already being collected under conventional field conditions in research and breeding programs. Traits that can be established as indicators of tolerance to warmer environments can then be validated across a larger set of genotypes without specifically screening in a warming experiment. For example, wheat is a long‐day plant, but over time breeders have selected for early maturity and thus PPD insensitivity. Our results suggest that photoperiod sensitivity might be a beneficial trait under a warmer climate. The preeminence of the PPD‐D1 locus means that incorporation of this trait would be straightforward (Jones et al., 2017).

In general, temperature increases are expected to accelerate development in wheat cultivars adapted to the southeastern United States regardless of photoperiod sensitivity. Previous research using infrared canopy warming, in both conventional and no‐tillage management, has suggested that the shift in phenology under warmed conditions is an adaptive response to maintain an adequate reproductive period and grain fill duration (Hou, Ouyang, Li, Wilson, & Li, 2012). Liu, Wang, Yang, and Wang (2010) reported that post‐flowering duration of wheat in China, over 25 years, remained stable despite a shortened vegetative period and overall shortened growing season, and linked this stability to varietal change overtime. Our study suggests that the rate of accelerated development under warming may be more dependent on specific environmental cues, such as daylength (photoperiod) for reproductive growth, or specifically the control of reproductive growth by the *Ppd‐D1* locus, allowing for a lengthened vegetative growing period.

Combining the knowledge that varietal improvement over time has aided in crop response to warming trends and that PPD sensitivity allows for lengthened vegetative growth, suggests that PPD‐sensitive varieties may be a management option to combat warmer temperatures. Further, these lines may escape risk for late spring freeze during critical periods of reproductive development in the southeastern United States, such as those observed in 2007, 2012, and 2017 (http://wheatscience.ca.uky.edu/newsletters; verified 14 May 2017). However, Hou et al. (2012) also suggest that an accelerated rate of development could also help wheat avoid higher temperatures at critical reproductive stages. In either scenario, the ability of a genotype to adapt to heat stress is largely linked to the ability to utilize assimilates efficiently under decreased grain fill duration or accelerated vegetative growth.

Photoperiod response is highly heritable (Ceccarelli et al., 2010) so coupling the PPD loci with other favorable traits such as resource‐use efficiencies (nutrient, water, radiation, etc.) may lead to favorable outcomes in plant breeding programs focused on climate change. Additionally, heat tolerance has been found to be proportional to the magnitude of temperature variation that an organism is exposed to during development. As such, crops like wheat and barley that are widely adapted to diverse thermal climates may contain greater genetic diversity for environmental stress tolerance (Addo‐bediako, Chown, & Gaston, 2000; Ceccarelli et al., 2010). Previous research has provided evidence for pleiotropic effects based on *Ppd‐1a* alleles (Jones et al., 2017). The influence of *Ppd‐1a* alleles on the initiation of flowering appears to be an important source of variation that could be tapped in selecting for resource‐use efficiency and the underlying adaptation to heat stress tolerance. Other research has demonstrated that the post‐anthesis development time is not the driver in adaptation to warmer environments and despite the shift in pre‐anthesis development, post‐anthesis development remains relatively unchanged (Tian et al., 2014).

Progress from selection depends on two factors: selection intensity and heritability (Falconer, 1960). Selection intensity can be very high, for example, selecting the top 1% of the population, but it will be for naught if the trait is not heritable, that is, if most of the variation measured is due to environmental rather than genetic factors. If an artificially warmed environment is to facilitate selection for genotypic adaptation to a warmer climate, then heritability of traits of interest must be of the same magnitude as estimates from the control environment. It was interesting that heritability of heading and anthesis dates (HDOM and ADOM), both considered high heritability traits in many crops, were higher in the warmed than in the control environment. Height, another high heritability trait, followed this same pattern.

In contrast to a passive warming companion study (Russell, 2017), the majority of the traits measured in the active warming study had higher heritabilities under warming (Table 5). Yield, which was essentially not heritable under passive warming had a moderately high heritability in the actively warmed environment. It was especially noteworthy that the traits related to NUE and N metabolism, generally had higher heritability estimates in the warmed environment than in the control.

Heritability often pertains just to the population in which the parameter is estimated, and with the caveat that these breeding lines and cultivars do not trace back to a single base population, these results offer encouragement to breeders who want to select in an artificially warmed environment. Caution is urged however, since we do not know at this point how well the results correspond to the real world warming that is occurring. Tian et al. (2014) observed across three years of warming on a single wheat cultivar a shortened pre‐anthesis developmental period, accompanied by significant increases in plant N uptake, total biomass accumulation, and grain yield. While Tian et al. (2014) found increased N concentrations in the leaf and stem at maturity under warming, we did not observe these trends. The lower vegetative N content at maturity in the warmed environment in our study is surprising considering the lack of significant differences in NUtE among environments, but may be attributed to the decreased number of spikes under warming.

Chapman et al. (2012) suggest when breeding for adaptation, the primary steps are to assess the potential impact of the environmental challenge and to identify traits for adaptation to this challenge. Active warming to screen for heat stress tolerance provides a resource‐efficient option for plant breeding programs in their initial assessment of genotypic performance in an artificially warmed environment. Having a baseline estimate of genotypic performance in a field setting allows plant breeders to make informed decisions about developing cultivars for variable climates. After identifying those genotypes that maintain or gain yield under increased temperatures, the next step will be to identify traits that confer this advantage. Ideally then one could develop a high‐throughput phenotyping method that would allow vast numbers of breeding lines to be screened. As more populations and breeding lines are genotyped with genomewide markers, the high‐throughput phenotyping could be coupled with genomic selection. With more accurate climate models in the future, the potential for genotype‐specific selection for growing season conditions may become a reality. Using data from in‐field trials, such as this one, allows for calibration of crop models linked to climate data.

## CONFLICT OF INTEREST

The authors report no conflict of interests.

## AUTHOR CONTRIBUTIONS

KGR conducted the research, analyzed the data, and wrote the manuscript. DVS helped plan the experiment, discussed data analysis, and edited and revised the manuscript.

## DATA ACCESSIBILITY

Data from this experiment will be stored at Figshare and will be publicly available.
